# Radiomics of multi-parametric MRI for the prediction of lung metastasis in soft-tissue sarcoma: a feasibility study

**DOI:** 10.1186/s40644-024-00766-9

**Published:** 2024-09-05

**Authors:** Yue Hu, Xiaoyu Wang, Zhibin Yue, Hongbo Wang, Yan Wang, Yahong Luo, Wenyan Jiang

**Affiliations:** 1 Department of Biomedical Engineering, China Medical University, Liaoning, 110122 China; 2 Department of Radiology, Liaoning Cancer Hospital and Institute, Liaoning, 110042 China; 3 Department of Radiology, Shengjing Hospital of China Medical University, Shenyang, 110004 China; 4 Department of Scientific Research and Academic, Liaoning Cancer Hospital and Institute, No. 44 Xiaoheyan Road, Liaoning, 110042 China; 5 Department of Radiology, The First Affiliated Hospital of Henan University of Traditional Chinese Medicine, Zhengzhou, 450000 China

**Keywords:** Soft-tissue sarcoma, Lung metastasis, MRI, Nomogram

## Abstract

**Purpose:**

To investigate the value of multi-parametric MRI-based radiomics for preoperative prediction of lung metastases from soft tissue sarcoma (STS).

**Methods:**

In total, 122 patients with clinicopathologically confirmed STS who underwent pretreatment T1-weighted contrast-enhanced (T1-CE) and T2-weighted fat-suppressed (T2FS) MRI scans were enrolled between Jul. 2017 and Mar. 2021. Radiomics signatures were established by calculating and selecting radiomics features from the two sequences. Clinical independent predictors were evaluated by statistical analysis. The radiomics nomogram was constructed from margin and radiomics features by multivariable logistic regression. Finally, the study used receiver operating characteristic (ROC) and calibration curves to evaluate performance of radiomics models. Decision curve analyses (DCA) were performed to evaluate clinical usefulness of the models.

**Results:**

The margin was considered as an independent predictor (*p < 0.05*). A total of 4 MRI features were selected and used to develop the radiomics signature. By incorporating the margin and radiomics signature, the developed nomogram showed the best prediction performance in the training (AUCs, margin vs. radiomics signature vs. nomogram, 0.609 vs. 0.909 vs. 0.910) and validation (AUCs, margin vs. radiomics signature vs. nomogram, 0.666 vs. 0.841 vs. 0.894) cohorts. DCA indicated potential usefulness of the nomogram model.

**Conclusions:**

This feasibility study evaluated predictive values of multi-parametric MRI for the prediction of lung metastasis, and proposed a nomogram model to potentially facilitate the individualized treatment decision-making for STSs.

**Supplementary Information:**

The online version contains supplementary material available at 10.1186/s40644-024-00766-9.

## Introduction

Soft tissue sarcomas (STSs) are a highly heterogeneous group of tumors with unpredictable clinical and pathological behaviors [1]. STSs can metastasize beyond the site of initial development (e.g., lung, bone, brain, liver, skin and rarely in lymph nodes) [2, 3]. Approximately 30–40% of STS patients may develop distant metastasis after excision of the primary tumor, which may significantly reduce patients’ life quality and survival rate [4, 5]. Clinical reports have demonstrated that the lung is the most frequent site of metastasis and accounts for around 90% of all metastatic cases [6]. Therefore, early and accurate detection of lung metastasis is crucial for making individual therapeutic decisions and improving prognosis in STSs.

In clinical practice, the fluoro-D-glucose (FDG) positron emission tomography (PET) and excisional biopsy are often required to obtain a definitive diagnosis of lung metastasis in STSs. However, the FDG-PET has a relatively high rate of false positive results, and may not be affordable for some patients [4]. The excisional biopsy requires significant expertise [7]. Inappropriate biopsy may cause serious consequences and has been considered as a major treatment error in management of STSs [8]. Magnetic resonance imaging (MRI) is noninvasive and has been widely used as a routine diagnostic tool in oncology. However, MRI can hardly provide accurate information regarding the nature of the soft tissue mass [9, 10]. Since there are many different subtypes of sarcomas that exhibit similar morphology on MRI [11], diagnoses based on MRI images by visual-assessments are usually suspected [12]. Therefore, clinical evaluation of the soft-tissue masses is still limited by subjective experiences of clinicians [13]. To our knowledge, there is still no specific MRI marker that has been evaluated for the preoperative prediction of lung metastasis in STSs.

In recent years, radiomics has been a rapidly growing discipline and considered as a promising and challenging field in cancer research, due to the capabilities of high-throughput analysis of quantitative features from medical images [14]. Numerous studies have highlighted values of radiomics in tumor diagnosis, prognosis and therapeutic response prediction [15, 16]. Radiomics has also been applied to reveal associations between MRI-based radiomics features and the underlying pathophysiology in soft-tissue tumor. However, previous studies mainly focused on the differentiation of benign and malignant soft-tissue tumors [17–19], the prediction of the development of distant metastasis [20] and histopathological grades [21–24] of the soft-tissue tumor. There have been reports that imaging features correlated to the LM status in STSs can be captured from FDG-PET [4, 25], T1-weighted and T2-weighted MRI [25, 26]. However, the studies both used data from the publicly available dataset (Cancer Imaging Archive, http://www.cancerimagingarchive.net/) and had inherent bias with limited number of samples, which may be because of sample collection challenges since the STS is a rare disease with low incidence [12]. A recently established deep learning model was evaluated with a larger sample size [27], but only focused on non-contrast-enhanced MRI. Inspired by these studies, we assumed that multi-parametric MRI-based radiomics may be predictive on the detection of lung metastasis in STSs, which to our knowledge has not been thoroughly studied. Thus, the goal of this study was to assess the predictive values of T1-CE and T2FS MRI-based radiomics, and explore a clinical-radiomics nomogram and evaluate its potential clinical usefulness.

## Materials and methods

### Patients

The retrospective study was approved by the institutional ethics committee of Liaoning Cancer Hospital and Institute. In total, 122 consecutive patients (mean age: 50; ranging from 19 to 82) were enrolled in the department of bone and soft tissue from Jul. 2017 and Mar. 2021 according to the following inclusion criteria: (i) pathologically confirmed with lung metastasis, (ii) complete T1-CE and T2FS MRI data before surgery, (iii) complete clinical data and histopathologic analysis of the primary resected soft-tissue mass and (iv) older than 18 years. Exclusion criteria were: (i) having other unrelated malignant oncology, (ii) incomplete clinical and/or imaging data, and (iii) underwent chemotherapy or radiotherapy before MRI scans. The patients were randomly divided into training and validation cohorts at a 2:1 ratio by stratified sampling. Baseline clinical characteristics, including age, gender, smoking, family history, past history, mobility, tenderness, hardness, size, margin, T1 signal matrix and T2 signal matrix were obtained from medical records in our hospital. Detailed descriptions of the clinical characteristics were available in Supplementary S1. Histologic information of all 122 STS patients was shown in Supplementary S2.

### MRI protocol and tumor segmentation

The STS patients underwent MRI scans with a Siemens 3.0T Verio MR scanner (Germany). The gadolinium-diethylenetriamine pentaacetic acid (Gd-DTPA) was used as contrast agent. The dose and speed of the Gd-DTPA injection were 0.1 mmol/kg and 3 ml/s, respectively. The T1-CE MRI scans were performed 30 s after the injection of the contrast agent. The parameters were: repetition time [TR]/ echo time [TE] = 700msec /11msec, slice thickness = 7 mm, and matrix size = 320 × 230. The fat-suppressed T2W MRI were acquired with the following parameters: TR/TE = 4130 msec/105 msec, slice thickness = 7 mm, and matrix size = 448 × 235. For each MRI modality, the regions of interests (ROIs) of the primary tumors on the MRI images were manually delineated by a radiologist with 12-year working experience (reader 1) along the border slice by slice using the ITK-SNAP v.3.6. The segmented ROIs were cross-validated by another radiologist (reader 2) with 7 years’ experiences.

### Radiomics feature extraction

For each MRI sequence, we calculated 1967 features from the whole tumor region using a “PyRadiomics” package in Python 3.6.0 as previously described [28]. The following three standardized feature classes were included: (i) first-order (*n* = 18 ), (ii) shape-based (*n* = 14) and (iii) textural features (*n* = 75), which quantitatively analyzed the heterogeneity of the image region based on the gray-level size-zone matrix (GLSZM, *n* = 16), gray-level run-length matrix (GLRLM, *n* = 16), gray-level co-occurrence matrix (GLCM, *n* = 24), gray level dependence matrix (GLDM, *n* = 14), and neighborhood gray-tone difference matrix (NGTDM, *n* = 5). Eight filters were applied to generate filtered images to obtain high-dimensional features. The filters include Wavelet, Laplacian of Gaussian, Logarithm, Exponential, Square, Gradient, LocalBinaryPattern2D/3D and SquareRoot. We extracted three standardized feature classes from original images, and the filtered images only generated first-order and textural features. Detailed information about radiomics features were described in the Pyradiomics documentation (url: http://pyradiomics.readthedocs.io) and in Supplementary S3.

### Feature selection

Radiomics features extracted from the T1-CE and T2FS MRI were combined to form a feature set with 3934 features. First, ROIs of thirty randomly selected STS patients (fifteen patients with LM and fifteen patients without LM) were resegmented by another radiologist (reader 2) with 7-year working experience to perform the interobserver intraclass correlation coefficient (ICC) analysis [29]. Features with ICCs higher than 0.85 were considered to have good consistency and remained. Next, applying Mann-Whitney *U* test to identify potential significant features between the lung metastasis and non-lung metastasis groups. Features with a *P* value < 0.05 were considered to be correlated to the LM status and remained. Afterwards, the least absolute shrinkage and selection operator (LASSO) was applied to further select features with 10-fold cross-validation for the selection of optimal λ using the “glmnet” package in R language (v.3.6.0) [30]. At last, in the logistic regression analysis, a stepwise selection was used to integrate the remained features with Akaike’s information criterion (AIC) as the stopping rule [31].

### Development of the radiomics signature and nomogram

The radiomics signature was developed based on the selected features weighted by their corresponding LASSO coefficients. The clinical-radiomics nomogram was constructed incorporating the radiomics signature and margin using the “rms” package in R. The ROC curve analysis was performed using the “pROC” package in R to evaluate the radiomics signature. The 95% confidence intervals (CI) were calculated by stratified bootstrap replicates using the “pROC” package. The diagnostic abilities of the proposed models were compared by DeLong test. Calibration curves were plotted using the “rms” package to assess the goodness-of-fit of the nomogram. Decision curve analysis (DCA) was implemented using the “rmda” package to calculate net benefits at different threshold probabilities to determine the clinical usefulness of the proposed models.

### Statistical analysis

Statistical analyses were performed to compare differences in clinical characteristics between LM and non-LM groups using the SPSS software (version 25.0). Continuous variables were performed with student’s *t* test or Mann-Whitney *U* test and fisher’s exact test or the $\:{\chi\:}^{2}$ test was performed on categorical variables, where appropriate. A two-sided *p* value < 0.05 was considered statistically significant.

## Results

### Clinical characteristics

Table 1 listed statistical analysis results of the clinical characteristics. The results showed that margin was significantly different between the lung metastasis and non-lung metastasis groups (*P* < 0.05). No significant difference was found between the two groups (*P* > 0.05) in regard to age, gender, smoking, family history, past history, mobility, tenderness, hardness, size, T1 signal matrix and T2 signal matrix. Stratified distributions of age were showed in Supplementary S4.

**Table 1:** Clinical characteristics of the STS patients

Characteristic	Training (*n* = 82)		*P*	Validation (*n* = 40)		*P*
	Non-lung metastasis (*n* = 47)	Lung metastasis (*n* = 35)		Non-lung metastasis (*n* = 23)	Lung metastasis (*n* = 17)	
Age (Mean ± SD)	50.28 ± 14.91	51.45 ± 16.11	0.274	50.62 ± 13.30	51.94 ± 16.57	0.685
Sex (%)			0.745			0.289
Male	32 (68.1)	25 (71.4)		11 (47.8)	11 (64.7)	
Female	15 (31.9)	10 (28.6)		12 (52.2)	6 (35.3)	
Family history (%)			1.000			1.000
Yes	4 (8.5)	2 (5.7)		1 (4.3)	0 (0.0)	
No	43 (91.5)	33 (94.3)		22 (95.7)	17 (100.0)	
Smoke history (%)			0.077			0.677
Yes	11 (23.4)	3 (8.6)		5 (21.7)	2 (11.8)	
No	36 (76.6)	32 (91.4)		18 (78.3)	15 (88.2)	
Past history (%)			0.634			0.471
Yes	34 (72.3)	24 (68.6)		16 (69.6)	14 (82.4)	
No	13 (27.7)	11 (31.4)		7 (30.4)	3 (17.6)	
Mobility (%)			0.563			0.730
Good	9 (19.1)	5 (14.3)		16 (69.6)	13 (76.5)	
Poor	38 (80.9)	30 (85.7)		7 (30.4)	4 (23.5)	
Tenderness (%)			0.844			1.000
Pain	34 (72.3)	26 (39.2)		17 (73.9)	12 (70.6)	
Non-pain	13 (38.3)	9 (60.8)		6 (26.1)	5 (29.4)	
Hardness (%)			0.943			0.683
Soft	5 (10.6)	3 (8.5)		1 (4.3)	0 (0.0)	
Tender	19 (40.4)	15 (42.9)		14 (60.9)	11 (64.7)	
Tough	23 (48.9)	17 (48.6)		8 (34.8)	6 (35.3)	
Size (%)			0.312			0.677
≥ 5 cm	43 (91.5)	29 (82.9)		18 (78.3)	15 (88.2)	
< 5 cm	4 (8.5)	6 (17.1)		5 (21.7)	2 (11.8)	
Margin (%)			0.027*			0.026*
Poor	30 (63.8)	30 (85.7)		14 (60.9)	16 (94.1)	
Well	17 (36.2)	5 (14.3)		9 (39.1)	1 (5.9)	
T1 signal matrix			0.983			0.672
Low	26 (55.3)	19 (54.3)		13 (56.5)	8 (47.1)	
Mixed	18 (38.3)	14 (40.0)		8 (34.8)	6 (35.3)	
High	3 (6.4)	2 (5.7)		2 (8.7)	3 (17.6)	
T2 signal matrix			0.432			0.707
Low	2 (4.3)	1 (2.9)		0 (0.0)	0 (0.0)	
Mixed	9 (19.1)	11 (31.4)		6 (26.1)	3 (17.6)	
High	36 (76.6)	23 (65.7)		17 (73.9)	14 (82.4)	

SD, standard deviation; *, *p* < 0.05

### Feature selection and development of the radiomics signature

**Fig. 1:**
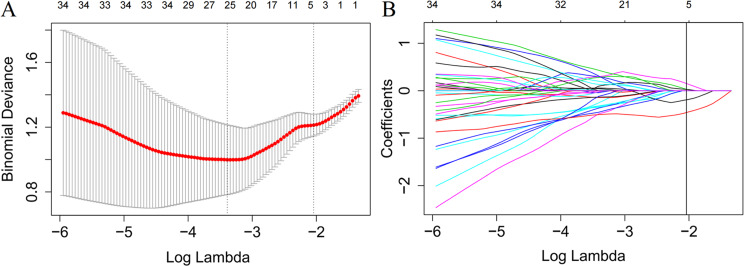
Feature selections from the multi-parametric MRI with LASSO. (**A**) Tuning parameter lambda selection in LASSO with 10-fold cross-validation. Vertical lines were drawn at the optimal values using the minimum criteria (left: the min criteria) and the 1 standard error of the minimum criteria (right: the 1-SE criteria). A λ value of 0.130, with log (λ), -2.040 was chosen (1-SE criteria). (**B**) LASSO coefficient profiles of the radiomics features, with 1-SE non-zero coefficients obtained from the MRI image. The vertical line was drawn at the optimal λ value which resulted in 5 non-zero coefficients

As shown in Fig. 1, radiomics features from two MRI sequences were selected through LASSO regression. In total, 4 features (2 from T1-CE and 2 from T2FS MRI) with the most predictive performance were finally used to develop the radiomics signature. Finally, we obtained the formula of the radiomics signature, weighted by the 4 features and their corresponding coefficients:

Radiomics signature = − 0.529–1.404 × original_shape_Sphericity + 0.942 × wavelet-HHL_glszm_SmallAreaEmphasis + 1.183 × lbp-3D-m2_glszm_ZoneEntropy − 0.841 × log-sigma-5-0-mm-3D_glszm_SmallAreaEmphasis.

**Fig. 2:**
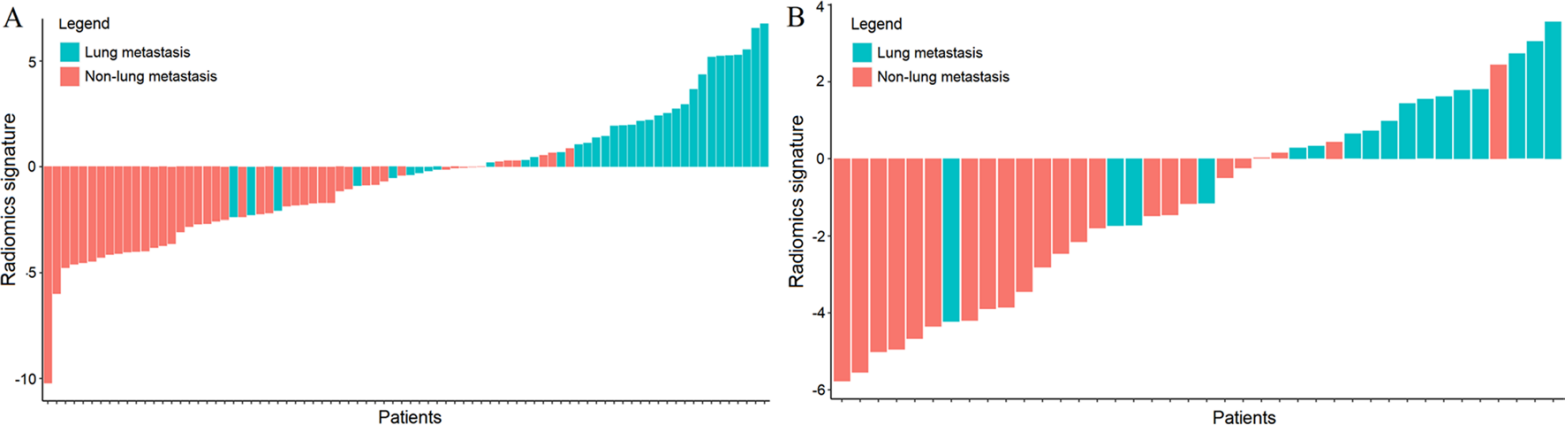
Bar charts of the radiomics signature for each patient in the training (**A**) and validation (**B**) cohorts. Red bars indicated STS patients in the non-lung metastasis group, and green bars indicated STS patients in the lung metastasis group

As shown in Fig. 2, the developed radiomics signature showed good performance to predict lung metastasis in STSs. The results indicated that patients with or without lung metastasis could be roughly discriminated since the lung metastasis group generally exhibited higher radiomics signature values than the non-lung metastasis group. Supplementary S5 showed T1-CE and T2FS MRI images of four patients, two are with high radiomics signature values and two are with low radiomics signature values. Comparisons of the performance of each selected feature were listed in Table 2. The Bland-Altman plots for the inter- and intra- reader variability of the selected radiomics features were shown in Supplementary S6 (Fig S3).

**Table 2:** Prediction performance of each radiomics feature

Feature	Source (MRI)	Cohort	Mean ± SD		AUC	*P*
			Non-lung metastasis	Lung metastasis		
Wavelet-HHL_glszm_SmallAreaEmphasis	T1-CE	Training	0.604 ± 0.047	0.646 ± 0.068	0.705	0.002
		Validation	0.617 ± 0.040	0.630 ± 0.069	0.501	0.497
Lbp-3D-m2_glszm_ZoneEntropy	T1-CE	Training	4.801 ± 0.447	5.170 ± 0.351	0.768	< 0.001
		Validation	4.668 ± 0.519	5.101 ± 0.353	0.772	0.003
Original_shape_Sphericity	T2FS	Training	0.674 ± 0.077	0.577 ± 0.079	0.811	< 0.001
		Validation	0.688 ± 0.071	0.635 ± 0.074	0.693	0.039
Log-sigma-5-0-mm-3D_glszm_SmallAreaEmphasis	T2FS	Training	0.653 ± 0.101	0.545 ± 0.122	0.739	< 0.001
		Validation	0.623 ± 0.110	0.541 ± 0.121	0.716	0.020

### Construction and validation of the nomogram

**Fig. 3:**
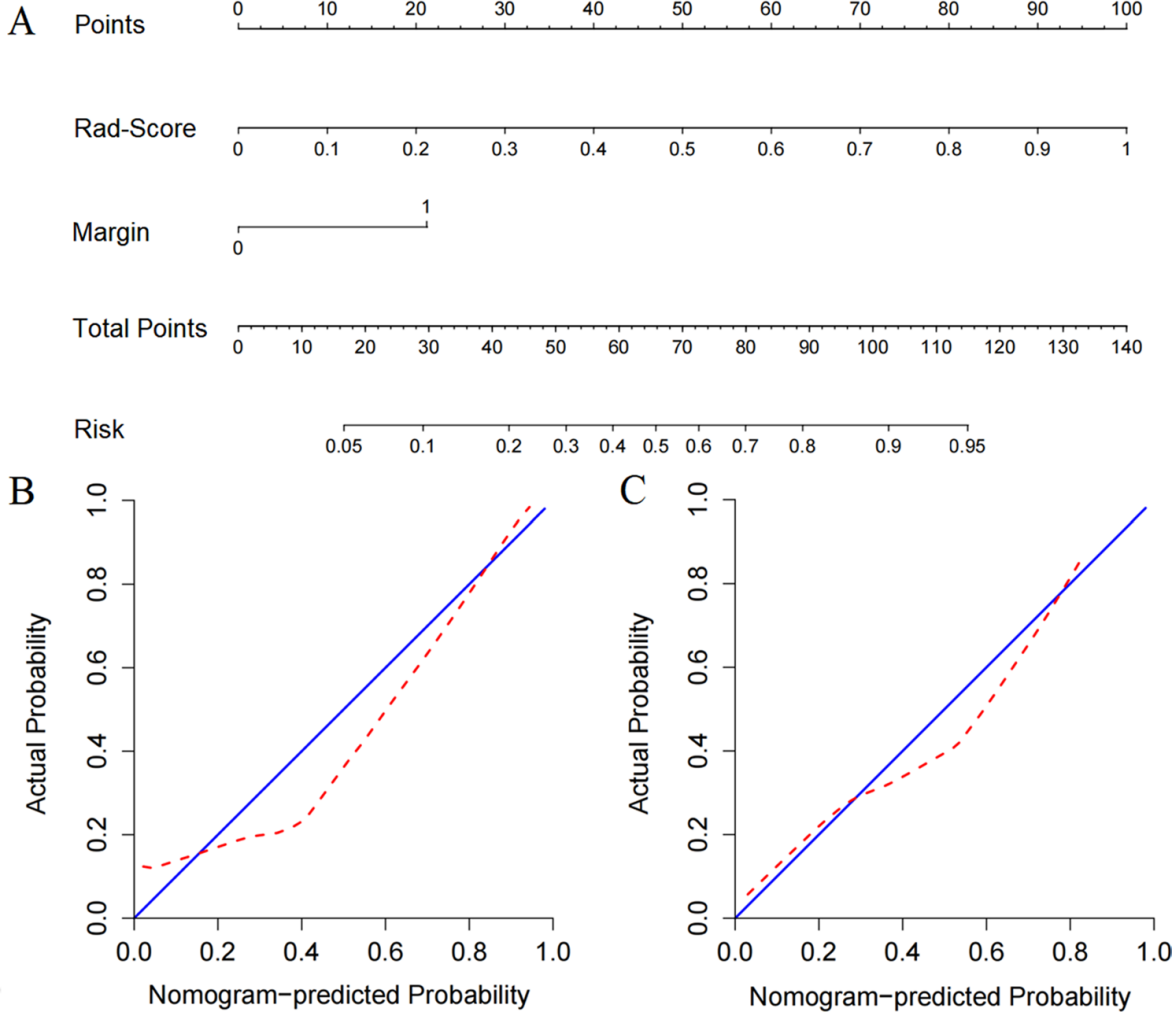
Construction and validation of the nomogram model. (**a**) The developed nomogram. (**b**) and (**c**) Calibration curves of the nomogram in the training (**b**) and validation (**c**) cohorts

**Fig. 4:**
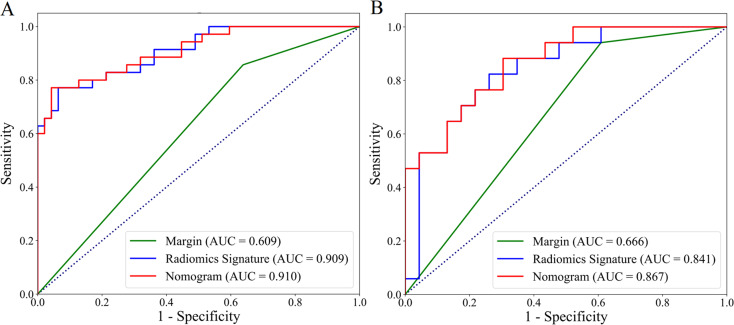
ROC curves of the margin, radiomics signature and nomogram in the training (**a**) and validation (**b**) cohorts

The margin was identified as important independent clinical predictor. The radiomics signature and margin were integrated to construct the clinical-radiomics nomogram. As shown in Fig. 3(A), the nomogram model was established with the radiomics signature in the second row, the margin in the third row, and total points in the fourth row. Calibration curves showed good calibration of the nomogram-predicted and actual values (Fig. 3(B) and 3(C)). Discriminating efficacies of the nomogram, radiomics signature and margin were proved in the ROC analysis (Fig. 4). The AUCs of the nomogram were 0.910 (95% [CI]: 0.847–0.974) for the training set and 0.894 (95%[CI]: 0.759–0.975) for the validation set. Comparing with the radiomics signature and margin, the nomogram achieved slightly better predictive performance with higher AUC and ACC values (Table 3). The Delong test results revealed significant differences between the margin and radiomics signature, and between the margin and nomogram (*P* > 0.05). The nomogram showed no significant improvement of the prediction performance (*P* = 0.909, 0.229 in the training and validation set, respectively) compared with the radiomics signature according to the Delong test.

**Table 3:** Comparisons of prediction performance of the radiomics signature, margin and nomogram

	Training cohort					Validation cohort				
	AUC (95% CI)	Acc	Spe	Sen	*P*	AUC (95% CI)	Acc	Sen	Sen	*P*
M1	0.909 (0.846–0.972)	0.817	0.936	0.771		0.841 (0.717–0.967)	0.750	0.739	0.824	
M2	0.609 (0.518–0.700)	0.573	0.638	0.857		0.666 (0.549–0.783)	0.575	0.609	0.941	
M3	0.910 (0.847–0.974)	0.841	0.957	0.771		0.894 (0.759–0.975)	0.775	0.696	0.882	
M1 vs. M2					< 0.001					0.044
M1 vs. M3					0.909					0.229
M2 vs. M3					< 0.001					0.006

M1, Radiomics signature; M2, Margin; M3, Nomogram; AUC, Area under curve; CI, Confidence interval; Acc, Accuracy; Sen, Sensitive; Spe, Specificity

**Fig. 5:**
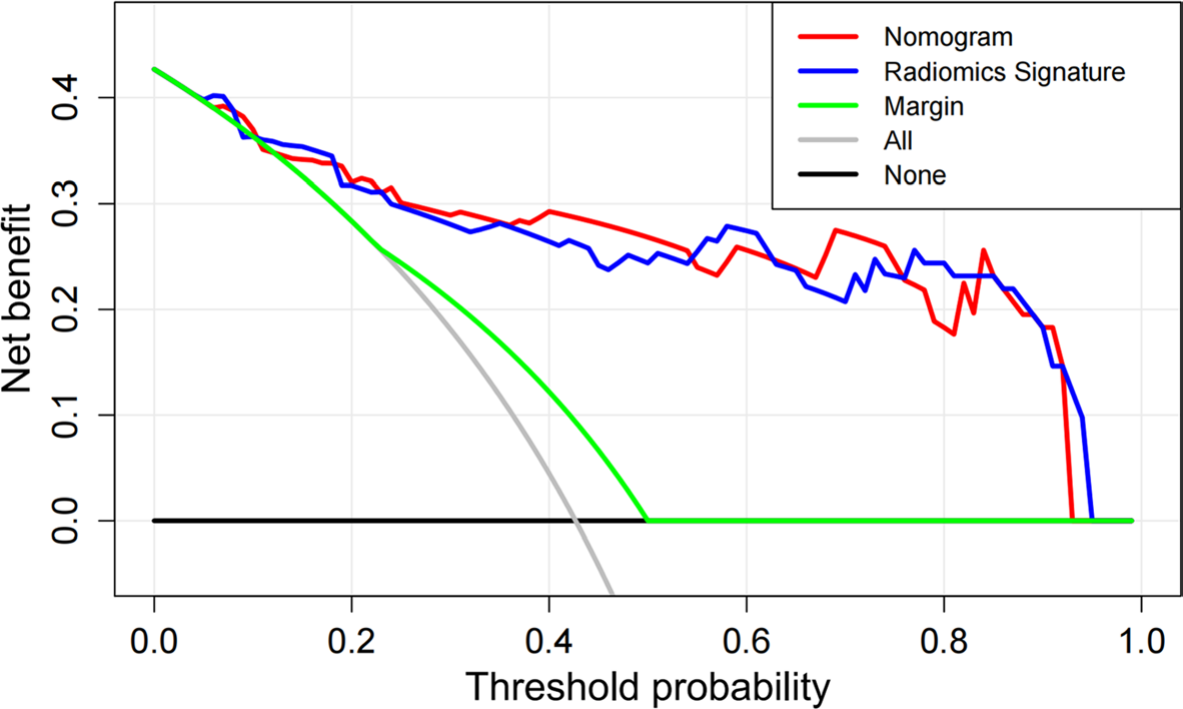
DCA curves of the margin, radiomics signature and nomogram. The x-axis represented the threshold probability, whereas the y-axis measured the net benefit for the patients. The black line represented the hypothesis that all patients were without lung metastasis. The gray line indicated the hypothesis that all patients were with lung metastasis. The red line represented the nomogram. The blue line represented the radiomics signature. The green line represented the margin

Figure 5 depicted DCA curves of the nomogram, radiomics signature and margin. The nomogram achieved the highest net benefit for the STSs among the three models when the threshold probability was between 0.05 and 0.92, which suggested good clinical potential of our proposed nomogram.

## Discussion

Lung metastasis (LM) is a crucial factor leading to poor prognosis in STSs, but only limited studies have explored values of preoperative imaging data on the prediction of LM [4, 25–27], mainly due to data collection challenges. Previous works only investigated correlations between non-contrast-enhanced MRI and the metastasis status [20, 27], and generated AUCs ranging from 0.799 to 0.902. In this study, we proposed a radiomics signature combing T1-CE and T2FS MRI, and generated AUCs of 0.909 and 0.841 in training and validation sets, respectively. This may indicate that the contrast-enhanced MRI can provide more information associated with the LM status.

A total of 3934 radiomics features were extracted and analyzed from the T1-CE and T2FS MRI, and 4 features were finally identified as the most predictive features associated with the lung metastasis. This was much more than previous studies related to our work that only evaluated 67 [4] and 50 [25] features in total. Among the 4 selected features, there were 3 features belong to the textural feature category (gray level size zone, glszm) and 1 feature belong to the shape-based feature category. The glszm feature quantifies gray level zones in an image and reflects heterogeneity and gray level changes within the tumor. Our results indicated that the intratumoral heterogeneity was highly associated with the lung metastasis. This was partially in line with a recent study that also found textural features from FDG-PET were associated with the lung metastasis in STS [4]. The original_shape_sphericity feature quantifies the roundness of the shape of the ROI relative to a circle. The bigger the value of the feature, the rounder the tumor. This indicated that tumors having more spherical shapes tend to be without LM, considering the average value of this feature in LM patients was smaller compared with those in non-LM patients. This was explainable since the rounder tumors may have lower degrees of malignancy, and have lower possibilities to metastasize.

Previous studies have indicated that some clinical features were predictive on the development of distant metastasis [32] and lung metastasis [4, 27] in STSs. However, the publish works only evaluated limited types of clinical factors and failed to evaluate tumor margin status. In this study, we comprehensively analyzed predictive values of clinical characteristics, and identified margin as the most predictive clinical factor. Although there is no previous report described relationships between margin and lung metastasis status, recent efforts suggested the margin was a discriminative factor for distinguishing benign from malignant soft tissue masses [18] and predicting histopathological grades of STSs [33]. This may be explainable by the fact that the margin reflected the conditions of the tumor edges, which was highly associated with the degree of malignancy, and hence was related to the metastasis status. We found that when used alone, the margin generated a lower AUC and accuracy, but higher sensitivity compared with the radiomics signature. This may be because of the overestimation of the degree of malignancy of the STS by using margin alone, due to the misjudgment of unclear peritumoral edema at the edges of the tumor. In our study, the factor age had no significant difference between the lung metastasis and non-lung metastasis groups (*P* > 0.05), which was consistent with previous reports by Liang et al. [27] and Tian et al. [20]. A previous study has considered age as a high-risk factor for the development of lung metastasis in STSs [4]. The reason for this discrepancy may be that their dataset came from different races and countries, which merits further investigations.

When margin was integrated with the radiomics signature, the constructed nomogram model showed slightly improvement in AUC and accuracy, which indicated that the margin could provide limited complementary information to radiomics features. The results generated by the nomogram were similar to those in a recent PET-based study for predicting the lung metastasis in STSs [4]. Calibration curves showed good agreements between our nomogram-predicted and actual results. The DCA indicated that the nomogram can obtain more net benefit compared with the radiomics signature or margin. Therefore, we suggested the proposed nomogram can be considered as a potential tool for the prediction of lung metastasis in STSs.

There were limitations in our work. First, the sample size was relatively small, because STS is a rare disease, and thus has sample collection challenges. Multi-center studies with a larger sample size should be conducted. Second, this study applied stratified sampling for dataset partition. The nested cross-validation [34] was reported to be capable of reducing the variations in the performance metrics across trails, and should be used in our future work. Third, a multi-modality study should be conducted with PET/CT and/or other MRI sequences combined to improve the predictive performance of the models. Fourth, molecular data (e.g. genes and proteins) were not included, which should be evaluated and integrated to the radiomics models to generate better results. Fifth, ROIs of the tumors were manually segmented, which was time-consuming and may lead to irregularity. Techniques such as automatic segmentation can be tried in our future research.

## Conclusions

This feasibility study evaluated values of multi-parametric MRI-based radiomics for predicting the lung metastasis in STSs, and presented a combined clinical-radiomics nomogram to potentially help clinicians guide treatment decisions.

## Electronic supplementary material

Below is the link to the electronic supplementary material.

Supplementary Material 1

## Data Availability

The data and material that support the findings of this study are available from the corresponding author upon reasonable request. The source code used in this study is available from https://github.com/CodeYueHu/Souce_code.

## Abbreviations

STS: Soft-tissue sarcoma
AUC: Area under the curve
ACC: Accuracy
T1-CE: T1-weighted contrast-enhanced
FDG-PET: Fluoro-D-glucose positron emission tomography
T2FS: T2-weighted fat-suppressed
MRI: Magnetic resonance imaging
ICC: Intraclass correlation coefficient
ROC: Receiver operating characteristic
DCA: Calibration and decision curve analysis
LASSO: Least absolute shrinkage and selection operator
AIC: Akaike information criterion
ROI: Region of interest
SEN: Sensitivity
SPE: Specificity
CI: Confidence interval
