# Supplementary material for: Radiomics of multi-parametric MRI for the prediction of lung metastasis in soft-tissue sarcoma: a feasibility study
Source: Cancer Imaging. 2024 Sep 5;24:119. doi: 10.1186/s40644-024-00766-9 (PMC11376009; doi:10.1186/s40644-024-00766-9)
Supplement: Supplementary file 1 — Supplementary Material 1 [file 40644_2024_766_MOESM1_ESM.docx]

Supplementary 1 **Clinical characteristics**

Since our study was retrospective, these clinical characteristics were derived from the electronic medical record system. MRI morphologic characteristics (size, margin, T1 signal matrix and T2 signal matrix) were reviewed by two radiologists with 12 and 7 years of musculoskeletal image diagnosis experience.

**Table S1.** Some of the clinical characteristic standards.

| **Clinical characteristics** | **Standards** | |
| --- | --- | --- |
| Mobility | Poor | The tumor is fixed and cannot be pushed. |
|  | Good | Tumor activity, which can be pushed on palpation. |
| Tenderness | Non-pain | The patient feels no pain when pressing. |
|  | Pain | The patient feels pain when pressed. |
| Texture | Tough | The tumor feels like a finger on the forehead. |
|  | Tender | The tumor feels like a finger touches the tip of the nose. |
|  | Soft | The tumor feels like a finger on the lip. |
| Margin | Ill-defined | The tumor tissue is not easy to distinguish from surrounding normal tissue, and there is no clear boundary between it and normal tissue. |
|  | Well-defined | The tumor tissue is significantly different from the surrounding normal tissue, or the tumor has a capsule. |
| Size | <5 cm | Maximum tumor diameter |
|  | ≥5 cm |  |

Supplementary 2 **Histologic types**

**Table S2**. Summary of the STS patients confirmed by histologic results.

| **LM (n = 67)** | **Number** | **Non-LM (n = 87)** | **Number** |
| --- | --- | --- | --- |
| Malignant fibrous histiocytomas | 16 | Malignant fibrous histiocytomas | 20 |
| Fibrosarcoma | 6 | Fibrosarcoma | 9 |
| Synovial sarcoma | 3 | Synovial sarcoma | 5 |
| Extraskelatal Ewing sarcoma | 3 | Extraskelatal Ewing sarcoma | 4 |
| Leiomyosarcoma | 10 | Leiomyosarcoma | 11 |
| Liposarcoma | 10 | Liposarcoma | 13 |
| Undifferentiated sarcoma | 2 | Undifferentiated sarcoma | 4 |
| Malignant tenosynovial giant cell tumor | 1 | Malignant tenosynovial giant cell tumor | 1 |
| Alveolar soft part sarcoma  Rhabdomyosarcoma  Extraskeletal myxoid chondrosarcoma  Extraskeletal osteosarcoma  Epithelioid sarcoma | 1  7  1  6  1 | Alveolar soft part sarcoma  Rhabdomyosarcoma  Extraskeletal myxoid chondrosarcoma  Extraskeletal osteosarcoma  Epithelioid sarcoma | 3  11  2  3  1 |

Supplementary 3 **Radiomics features**

**Table S3**. Detailed descriptions of the radiomics features.

| **Non-texture features (31)** | |
| --- | --- |
| **Feature type** | **Feature name** |
| **Shape-based features (14)** | Elongation |
|  | Flatness |
|  | Least Axis Length |
|  | Major Axis Length |
|  | Maximum 2D diameter (Column) |
|  | Maximum 2D diameter (Row) |
|  | Maximum 2D diameter (Slice) |
|  | Maximum 3D diameter |
|  | Mesh Volume |
|  | Minor Axis Length |
|  | Sphericity |
|  | Surface Area |
|  | Surface Area to Volume ratio |
|  | Voxel Volume |
| **First order features (17)** | 10th percentile |
|  | 90th percentile |
|  | Energy |
|  | Entropy |
|  | Interquartile Range |
|  | Kurtosis |
|  | Maximum |
|  | Mean |
|  | Mean Absolute Deviation |
|  | Median |
|  | Minimum |
|  | Range |
|  | Robust Mean Absolute Deviation |
|  | Root Mean Squared |
|  | Skewness |
|  | Total Energy |
|  | Uniformity |
|  | Variance |
| **Texture features (75)** | |
| **Feature type** | **Feature name** |
| **GLCM features (24)** | Autocorrelation |
|  | Cluster Prominence |
|  | Cluster Shade |
|  | Cluster Tendency |
|  | Contrast |
|  | Correlation |
|  | Difference Average |
|  | Difference Entropy |
|  | Difference Variance |
|  | Inverse Difference |
|  | Inverse Difference Moment |
|  | Inverse Difference Moment Normalized |
|  | Inverse Difference Normalized |
|  | Informational Measure of Correlation (IMC) 1 |
|  | Informational Measure of Correlation (IMC) 2 |
|  | Inverse Variance |
|  | Joint Average |
|  | Joint Energy |
|  | Joint Entropy |
|  | Maximal Correlation Coefficient |
|  | Maximum Probability |
|  | Sum Average |
|  | Sum Entropy |
|  | Sum of Squares |
| **GLDM features (14)** | Dependence Entropy |
|  | Dependence Non-Uniformity |
|  | Dependence Non-Uniformity Normalized |
|  | Dependence Variance |
|  | Gray Level Non-Uniformity |
|  | Gray Level Variance |
|  | High Gray Level Emphasis |
|  | Large Dependence Emphasis |
|  | Large Dependence High Gray Level Emphasis |
|  | Large Dependence Low Gray Level Emphasis |
|  | Low Gray Level Emphasis |
|  | Small Dependence Emphasis |
|  | Small Dependence High Gray Level Emphasis |
|  | Small Dependence Low Gray Level Emphasis |
| **GLRLM features (16)** | Gray Level Non-Uniformity |
|  | Gray Level Non-Uniformity Normalized |
|  | Gray Level Variance |
|  | High Gray Level Run Emphasis |
|  | Long Run Emphasis |
|  | Long Run High Gray Level Emphasis |
|  | Long Run Low Gray Level Emphasis |
|  | Low Gray Level Run Emphasis |
|  | Run Entropy |
|  | Run Length Non-Uniformity |
|  | Run Length Non-Uniformity Normalized |
|  | Run Percentage |
|  | Run Variance |
|  | Short Run Emphasis |
|  | Short Run High Gray Level Emphasis |
|  | Short Run Low Gray Level Emphasis |
| **GLSZM features (16)** | Gray Level Non-Uniformity |
|  | Gray Level Non-Uniformity Normalized |
|  | Gray Level Variance |
|  | High Gray Level Zone Emphasis |
|  | Large Area Emphasis |
|  | Large Area High Gray Level Emphasis |
|  | Large Area Low Gray Level Emphasis |
|  | Low Gray Level Zone Emphasis |
|  | Size-Zone Non-Uniformity |
|  | Size-Zone Non-Uniformity Normalized |
|  | Small Area Emphasis |
|  | Small Area High Gray Level Emphasis |
|  | Small Area Low Gray Level Emphasis |
|  | Zone Entropy |
|  | Zone Percentage |
|  | Zone Variance |
| **NGTDM features (5)** | Busyness |
|  | Coarseness |
|  | Complexity |
|  | Contrast |
|  | Strength |

Detail descriptions for each filter were as following:

(1) **Wavelet filtering**: Decomposes the images into high-frequency components (H) or low-frequency components (L) at the three directions. Eight categories of wavelet features were acquired and labeled as **HHH, HHL, HLH, LHH, LLL, LLH, LHL, HLL** based on different decomposition orders.

(2) **LoG filtering**: Laplacian of Gaussian filter (**sigma = 1.0, 3.0, 5.0**) defines how coarse the emphasised texture should be. Three categories of log features were acquired and labeled as sigma 1.0, sigma 3.0, sigma 5.0 based on different values of sigma.

(3) **Square filtering**: Takes the square of the image intensities and linearly scales them back to the original range.

(4) **SquareRoot filtering**: Takes the square root of the absolute image intensities and scales them back to original range.

(5) **Logarithm filtering**: Takes the logarithm of the absolute intensity + 1.

(6) **Exponential filtering**: Takes the the exponential, where filtered intensity is e^ (absolute intensity).

(7) **Gradient filtering**: Returns the magnitude of the local gradient.

(8) **Localbinarypattern filtering**: Localbinarypattern 2D/3D, including **lbp-2D, lbp-3D-k, lbp-3D-m1, lbp-3D-m2**. LocalBinaryPattern2D: calculates and returns a local binary pattern applied in 2D. LocalBinaryPattern3D: calculates and returns local binary pattern maps applied in 3D using spherical harmonics. Last returned image is the corresponding kurtosis map.

Supplementary 4 **Stratified distributions of age**


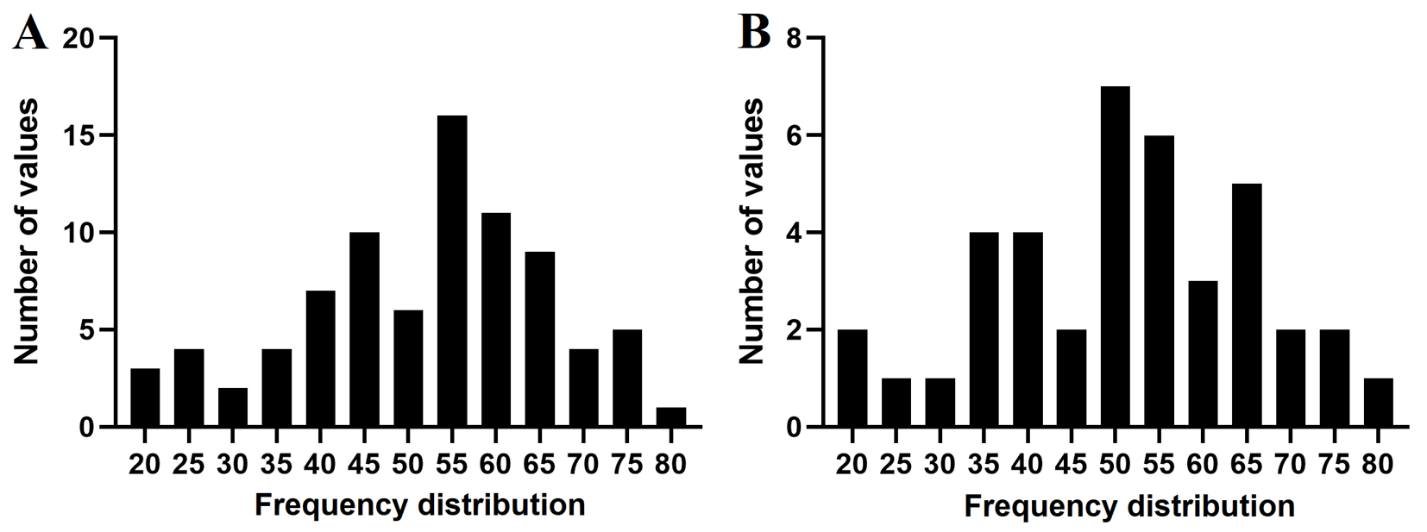


**Fig S1.** Histograms of the age in the training (A) and validation (B) sets.

Supplementary 5 **Patients with high or low signature values**

Fig S2 showed T1-CE and T2FS MRI images of 4 patients (2 with high radiomics signature values and 2 with low radiomics signature value).


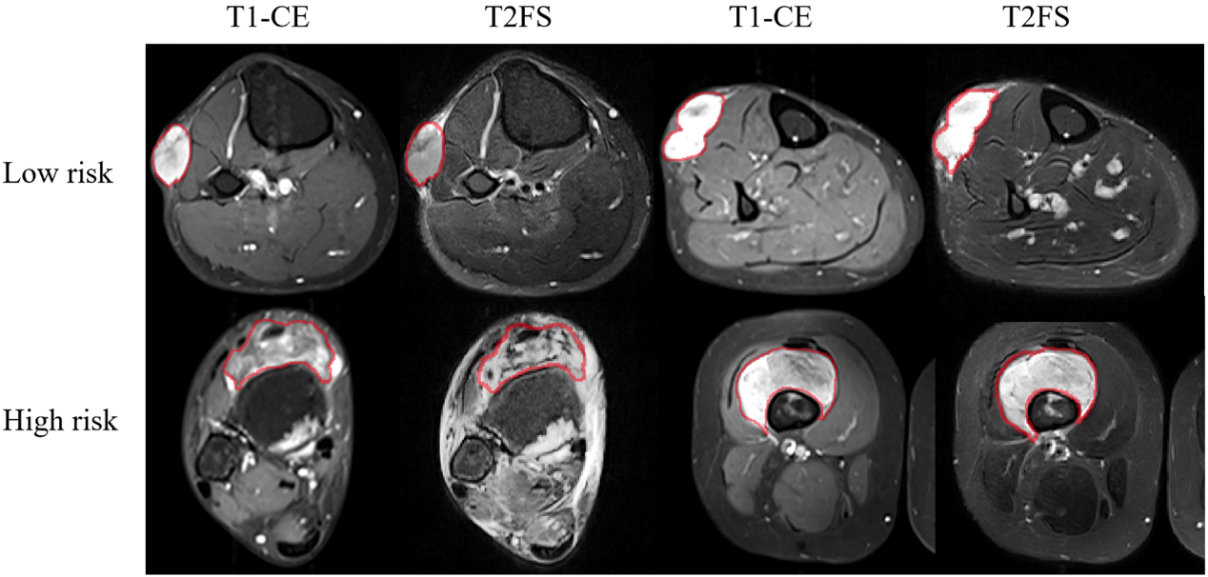


**Fig S2.** The top row showed MRI images with low radiomics signature values, and the bottom row showed images with high radiomics signature values, both containing T1-CE and T2FS sequences.

Supplementary 6 **Bland-Altman plots**


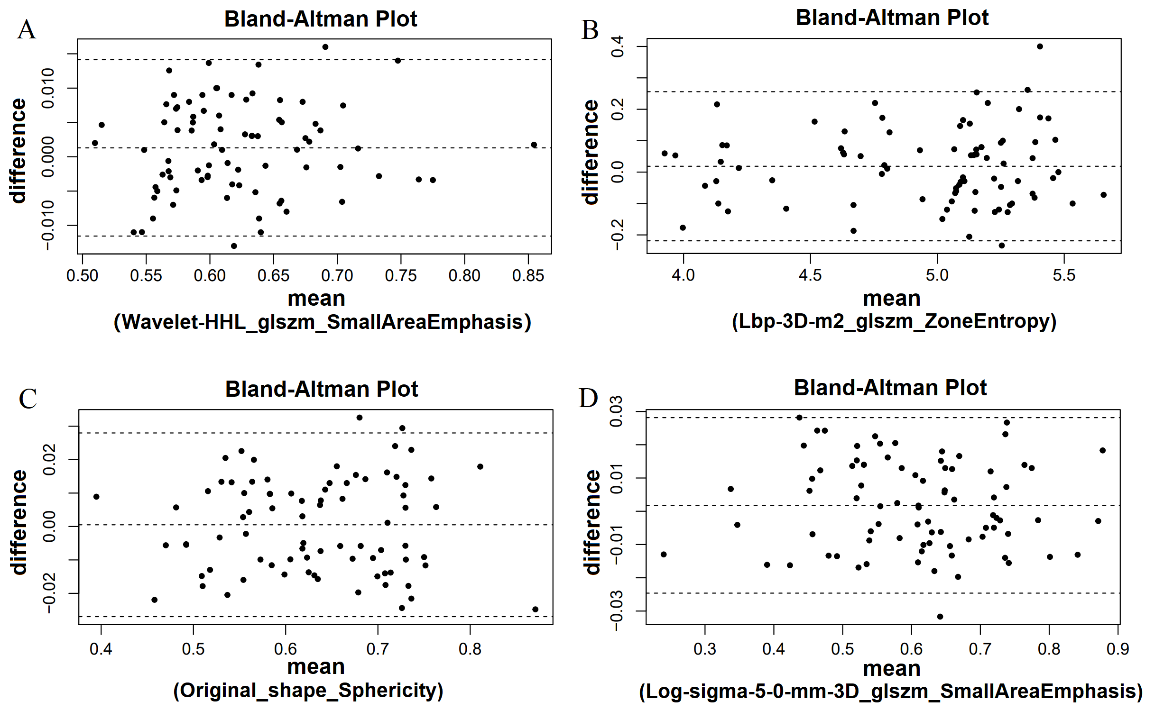


**Fig S3.** Bland-Altman analysis for the selected radiomics features. (A) Wavelet-HHL_glszm_SmallAreaEmphasis, (B) Lbp-3D-m2_glszm_ZoneEntropy, (C) Original_shape_Sphericity, (D) Log-sigma-5-0-mm-3D_glszm_SmallAreaEmphasis. Dashed lines denoted mean and 95% confidence interval of differences of the features, respectively.
